# Category clustering calculator for free recall

**DOI:** 10.2478/v10053-008-0124-y

**Published:** 2012-11-16

**Authors:** Olesya Senkova, Hajime Otani

**Affiliations:** Department of Psychology, Central Michigan University, USA

**Keywords:** free recall, category clustering

## Abstract

The free recall measure is one of the most popular measures in memory research. Using this measure, researchers can assess not only the amount of recall but also the strategy participants used to recall the material. *Category clustering* is a strategy participants often use when the input list is categorized. Unfortunately, computing category clustering measures is laborious. The present paper introduces a calculator that computes these measures for each participant using a platform that is accessible to most researchers in an attempt to make these measures more user-friendly.

The free recall measure has been the workhorse of memory research ever since the popularity of the paired-associates learning paradigm has declined due to the adoption of the information processing approach by many psychologists. As of September 4, 2012, a PsychINFO® database (http://www.apa.org/pubs/databases/psycinfo/index.aspx; American Psychological Association) search using a keyword free recall resulted in 7,020 hits. These published studies cover research in almost every area of psychology. One of the strengths of the *free recall* measure is simplicity; no special equipment is needed. Participants are asked to reproduce as many of the to-be-remembered (TBR) items as possible (in any order) and respond either orally or by writing their responses. Researchers then count the number of correct items as well as incorrect items (intrusions). The free recall measure, however, has an additional advantage of revealing participants’ strategy to organize the TBR material. That is, although two participants may have recalled the same number of items, one participant may have organized the material based on taxonomic categories whereas another participant may not have used any particular organizational scheme. A question can also be asked as to the reason that one group of participants (e.g., patient group, Group A) shows memory impairment whereas the other group (control group, Group B) does not. A possible answer is that Group A is suffering from a condition that causes impairment in the ability to actively organize the TBR materials (cf. e.g., BrØnnick et al., 2011).

An obvious way of organizing the TBR materials (e.g., a list of words) is to group items that belong to the same taxonomic category (e.g., fruit) and output these items together. This strategy, referred to as *category clustering*, was identified by W. A. Bousfield (1953) and has been shown to be a robust strategy used by participants when the input list is categorized. A number of measures have been created to identify the extent of category clustering: RR (Ratio of Repetition; Cohen, Sakoda, & Bousfield, 1954), MRR (Modified Ratio of Repetition; Wallace & Underwood, 1964), DS (Deviation Score; A. K. Bousfield & Bousfield, 1966), ARC (Adjusted Ratio of Clustering; Gerjuoy & Spitz, 1966; Roenker, Thompson, & Brown, 1971). However, researchers who are interested in using these measures are often discouraged by the daunting task of going over each participant’s recall protocol and hand calculating these measures. There have been past attempts to create computer programs to make the computation less laborious (e.g., Elie & Payne, 1999; Kazen & Otani, 1997); however, the platforms these programs were based on (e.g., a mainframe computer version of SPSS, 1988) are now obsolete. We, therefore, decided to create a calculator using a platform that is accessible to almost anyone using a computer, Microsoft® Excel (Microsoft Corporation). Our goal is to make these measures more user-friendly in hope that researchers would be more likely to use these measures to take advantage of rich data provided by the free recall measure.

The calculator is designed to compute the measures mentioned above (RR, MRR, DS, and ARC) for each participant. The formulae we used to derive these measures are listed in Table 1 (adopted from Murphy & Puff, 1982). The calculator computes these measures in two ways: (a) using all recalled items and (b) using correctly recalled items only. Data input is fairly simple; code each item (e.g., *apple*) as either correct (1) or incorrect (0) and input these in the serial order of recall output. Then, for each item, input a code that represents a particular category (e.g., 1 for fruit). (For an example experiment and the scoring instructions, see Appendix A.) The output includes two tables that display the computed values in a column. However, to make it easier to cut and paste these values into statistical software, the third table displays these values in a row. Currently, the calculator is set to handle up to 1,000 items with up to eight categories; however, it can be easily modified to handle as many items and categories as one would need. Note, however, that there is a limit as to how many items as well as categories one can free recall. The accuracy of the calculator was verified by using eight mock datasets shown in Table 2. Table 3 shows the measures computed based on all recalled items or correctly recalled items only. The values computed by the calculator matched those computed by hand-calculation. The calculator (cmcc.xlsx) and the scoring instructions can be obtained by downloading these files from the following website (http://otanicognitionlab.weebly.com/resources.html) or by e-mailing a request to Hajime Otani at Central Michigan University, Michigan, USA (otani1h@cmich.edu).

**Table 1.** Parameters and Formula Used to Compute RR, MRR, DS, and ARC

*n* = number of recalled items
*c* = number of recalled categories
*r* = number of category repetition
*n_i_* = number of items recalled in each recalled category
max = *n* − *c*
*E*(*r*) = Σ *n_i_*^2^/*n* − 1

RR = *r*/(*n* − 1)
MRR = *r*/max
DS = *r* − *E*(*r*)
ARC = [*r* − *E*(*r*)]/[max − *E*(*r*)]

*Note*. ARC = Adjusted Ratio of Clustering. DS = Deviation Score. MRR = Modified Ratio of Repetition. RR = Ratio of Repetition. Adapted from “Free Recall: Basic Methodology and Analysis,” by M. D. Murphy and C. R. Puff, in *Handbook of Research Methods in Human Memory and Cognition*, by C. R. Puff (Ed.), 1982, New York: Academic Press, p. 120.

**Table 2.** Mock Datasets Used to Verify the Accuracy

	Participant							
Output order	A	B	C	D	E	F	G	H
1	1 2	1 3	1 4	1 2	1 2	1 3	1 2	1 3
2	1 4	1 4	1 4	1 1	1 3	1 3	1 2	1 4
3	1 4	1 4	1 4	1 3	1 2	1 3	1 3	1 4
4	1 3	1 3	1 4	1 2	1 4	1 4	1 1	1 3
5	1 2	1 1	1 1	1 2	1 4	1 4	1 1	1 3
6	1 3	1 1	1 1	1 2	1 2	1 2	1 1	1 1
7	1 1	1 3	1 2	1 1	1 2	1 3	1 1	1 1
8	1 4	1 1	1 3	1 4	1 1	1 1	1 2	1 4
9	1 4	1 1	1 3	1 4	1 3	1 1	1 3	1 2
10		1 2	1 3		1 3	1 1	1 3	0 3
11		1 2	0 3		1 3	1 2	1 2	0 4
12		1 2	1 2		1 4	1 2	1 1	
13		1 4	1 2		1 4	1 2	1 4	
14		1 4	1 1		1 4	1 4	1 4	
15		1 3	1 2		1 3	1 4	1 4	
16					0 4	1 4	1 4	
17					1 1		1 2	
18							1 2	
19							1 3	
20							0 1	

*Note*. For each participant, the first column indicates whether each recalled item is correct (1) or incorrect (0) whereas the second column indicates to which category each item belong (1, 2, 3, 4).

**Table 3.** Measures Computed Based on Mock Datasets

Measures	Participants							
	A	B	C	D	E	F	G	H
*n*	9	15	15	9	17	16	20	11
*c*	4	4	4	4	4	4	4	4
*r*	2	6	8	3	6	9	9	3
Max	5	11	11	5	13	12	16	7
*E(r)*	1.78	2.80	2.80	1.78	3.76	3.12	4.20	2.36
RR	.25	.43	.57	.38	.38	.60	.47	.30
MRR	.40	.55	.73	.60	.46	.75	.56	.43
DS	0.22	3.20	5.20	1.22	2.24	5.88	4.80	0.64
ARC	.07	.39	.63	.38	.24	.66	.41	.14
n*			14		16		19	9
c*			4		4		4	4
r*			7		6		9	3
max*			10		12		15	5
E(r)*			2.57		3.38		3.89	1.56
RR*			.54		.40		.50	.38
MRR*			.70		.50		.60	.60
DS*			4.43		2.62		5.11	1.44
ARC*			.60		.30		.46	.42

*Note*. ARC = Adjusted Ratio of Clustering. DS = Deviation Score. MRR = Modified Ratio of Repetition. RR = Ratio of Repetition. The measures without asterisks were computed based on all recalled items whereas the measures with asterisks were computed based on correctly recalled items only. In the case where there was no incorrect recall, only the formers are shown.

## Figures and Tables

**Table A4.**
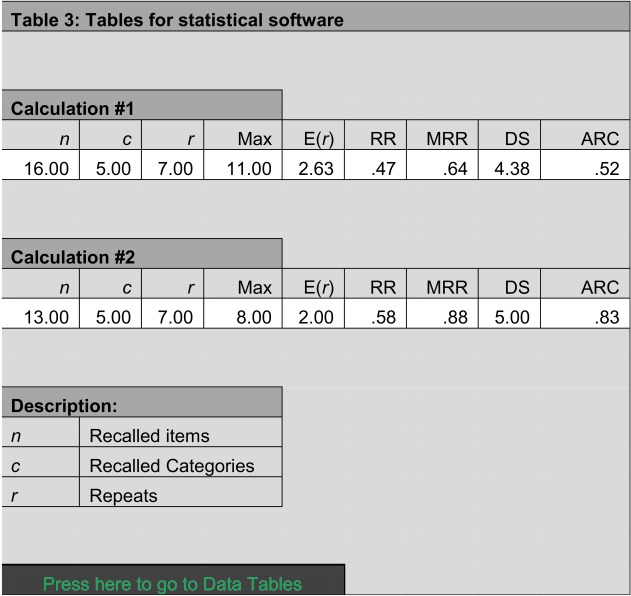
Computed Values from the Above Example (Table 3 of the Calculator)

## Appendix A Example experiment and scoring instructions

An experiment was conducted using a list that consisted of six nouns from each of six taxonomic categories. These nouns were presented in a random order (see Table A1), and participants were asked to remember as many words as possible. Following the presentation, participants performed a filler task (a simple arithmetic task) for 2 min. Then, they completed a free recall test in which they wrote as many of the words from the study list as possible in any order. The investigator was interested in two measures: (a) the number of correct responses and (b) the degree of category clustering.

The first step in scoring the output for this participant is to code each response in terms of (a) whether the response is correct (1– correct, 0 – incorrect) and (b) which category out of six categories the item was from (1– body, 2 – weapon, 3 – weather, 4 – building, 5 – animal, or 6 – vegetable ). Table A3 shows coding example.

The next step is to input the scored output in the Category Clustering Calculator. Input the third and seventh column of Table 3 (labeled *Correct*) to the column labeled *Recalled items* of the Calculator. Input the fourth and eighth column of Table 3 (labeled *Category*) to the column labeled *Recalled items* of the Calculator. Be sure to maintain the output order of the recalled items.

The Calculator will compute the following clustering measures for this participant, RR (Ratio of Repetition), MRR (Modified Ratio of Repetition), DS (Deviation Score), and ARC (Adjusted Ratio of Clustering), along with *n* (number of recalled items), *c* (number of recalled categories), and *r* (number of category repetition). There will be two outputs: (a) the output based on all recalled items and (b) the output based on correctly recalled items only. Next, cut and paste the output into statistical software, such as SPSS. Because statistical software often uses a row to represent each participant, Table 3 of the Calculator display the computed values in a row (see Table A4). Highlight the row and paste it into statistical software.

**Table A1.** Study List Consisting of Six Nouns from Six Taxonomic Categories

1	*rain*	10	*ceiling*	19	*potato*	28	*camel*
2	*bear*	11	*window*	20	*harpoon*	29	*knife*
3	*rib*	12	*sward*	21	*beaver*	30	*frost*
4	*stair*	13	*horse*	22	*bomb*	31	*thunder*
5	*monsoon*	14	*hatchet*	23	*tomato*	32	*ear*
6	*rifle*	15	*toe*	24	*fog*	33	*cucumber*
7	*goat*	16	*roof*	25	*bean*	34	*snow*
8	*corn*	17	*lettuce*	26	*shoulder*	35	*waist*
9	*nose*	18	*dog*	27	*wall*	36	*floor*

**Table A2.** Recall Output of Participant A

1	*shoulder*	7	*potato*	13	*horse*
2	*ear*	8	*corn*	14	*carrot*
3	*nose*	9	*cucumber*	15	*cat*
4	*rifle*	10	*goat*	16	*door*
5	*knife*	11	*bear*		
6	*tomato*	12	*window*		

**Table A3.** Scored Recall Output for Participant A

	Item	C	CT		Item	C	CT
1	*shoulder*	1	1	9	*cucumber*	1	6
2	*ear*	1	1	10	*goat*	1	5
3	*nose*	1	1	11	*bear*	1	5
4	*rifle*	1	2	12	*window*	1	4
5	*knife*	1	2	13	*horse*	1	5
6	*tomato*	1	6	14	*carrot*	0	6
7	*potato*	1	6	15	*cat*	0	5
8	*corn*	1	6	16	*door*	0	4

*Note*. C = correct. CT = category.
